# Beneficial Effects of Physical Exercise on Functional Capacity and Skeletal Muscle Oxidative Stress in Rats with Aortic Stenosis-Induced Heart Failure

**DOI:** 10.1155/2016/8695716

**Published:** 2016-01-20

**Authors:** Mariana Janini Gomes, Paula Felippe Martinez, Dijon Henrique Salomé Campos, Luana Urbano Pagan, Camila Bonomo, Aline Regina Ruiz Lima, Ricardo Luiz Damatto, Marcelo D. M. Cezar, Felipe Cezar Damatto, Camila Moreno Rosa, Camila Marchiolli Garcia, David Rafael Abreu Reyes, Ana Angélica Henrique Fernandes, Denise Castro Fernandes, Francisco Rafael Laurindo, Katashi Okoshi, Marina Politi Okoshi

**Affiliations:** ^1^Botucatu Medical School, Internal Medicine Department, Sao Paulo State University (UNESP), Botucatu, SP, Brazil; ^2^Institute of Biosciences of Botucatu, Sao Paulo State University (UNESP), Botucatu, SP, Brazil; ^3^Heart Institute, School of Medicine, University of Sao Paulo (USP), Sao Paulo, SP, Brazil

## Abstract

*Objective*. We evaluated the influence of exercise on functional capacity, cardiac remodeling, and skeletal muscle oxidative stress, MAPK, and NF-*κ*B pathway in rats with aortic stenosis- (AS-) induced heart failure (HF).* Methods and Results*. Eighteen weeks after AS induction, rats were assigned into sedentary control (C-Sed), exercised control (C-Ex), sedentary AS (AS-Sed), and exercised AS (AS-Ex) groups. Exercise was performed on treadmill for eight weeks. Statistical analyses were performed with Goodman and ANOVA or Mann-Whitney. HF features frequency and mortality did not differ between AS groups. Exercise improved functional capacity, assessed by maximal exercise test on treadmill, without changing echocardiographic parameters. Soleus cross-sectional areas did not differ between groups. Lipid hydroperoxide concentration was higher in AS-Sed than C-Sed and AS-Ex. Activity of antioxidant enzymes superoxide dismutase and glutathione peroxidase was changed in AS-Sed and restored in AS-Ex. NADPH oxidase activity and gene expression of its subunits did not differ between AS groups. Total ROS generation was lower in AS-Ex than C-Ex. Exercise modulated MAPK in AS-Ex and did not change NF-*κ*B pathway proteins.* Conclusion*. Exercise improves functional capacity in rats with AS-induced HF regardless of echocardiographic parameter changes. In soleus, exercise reduces oxidative stress, preserves antioxidant enzyme activity, and modulates MAPK expression.

## 1. Introduction

Heart failure is an important public health issue due to its high prevalence and poor prognosis [1]. Heart failure is clinically characterized by a reduced exercise capacity with the early occurrence of fatigue and dyspnea [2, 3]. Skeletal muscle abnormalities can contribute to the impaired ability to perform physical activities. Muscle changes include wasting and fibrosis, changes in fiber type and myosin heavy chain composition, decrease in oxidative capacity, increase in oxidative stress, and contractile dysfunction [4–8].

Current guidelines strongly recommend regular physical exercise for patients with stable heart failure to prevent and/or attenuate cardiac remodeling and skeletal muscle alterations [9, 10]. Clinical and experimental studies have shown that physical exercise attenuates abnormal cardiac remodeling, reduces muscle wasting and local inflammation, improves muscle capillarization, blood flow, and oxygen utilization, and increases functional capacity, exercise duration, and quality of life [11–16].

However, most studies on the effects of exercise have evaluated postmyocardial infarction-induced heart failure. The influence of exercise during conditions characterized by persistent left ventricular pressure-overload such as aortic stenosis remains unsettled. As life expectancy is increasing and aortic stenosis prevalence augments with age, the number of patients with pressure-overload-induced heart failure will grow [17]. Experimental studies on the effects of exercise on cardiac and skeletal muscle during aortic stenosis have shown controversial results. Mice with mild or severe aortic stenosis subjected to short-term voluntary rotating wheel exercise presented unchanged left ventricular function with a trend towards aggravated ventricular dysfunction in severe cases [18]. On the other hand, we observed that aerobic treadmill training attenuated systolic dysfunction during transition from compensated cardiac dysfunction to heart failure in aortic stenosis rats [19]. Furthermore, exercise prevented skeletal muscle atrophy through anticatabolic effects [19].

Ascending aortic stenosis in rats has been used to induce persistent and chronic pressure overload. In this model, 3-4 week-old rats are subjected to a clip placement around the ascending aorta. After clip placement, aorta diameter is preserved; as rats grow, stenosis progressively develops. The model has the advantage that, despite rapid left ventricular hypertrophy onset, ventricular dysfunction and heart failure occur slowly [19], similar to what is observed in human chronic pressure overload. In this study we evaluated the influence of physical exercise on functional capacity, cardiac remodeling, and skeletal muscle phenotype and oxidative stress in rats with aortic stenosis-induced heart failure. Since the NADPH oxidase (NOX) family is an important source of reactive oxygen species in various tissues [20] and skeletal muscle expresses two NADPH oxidase isoforms, we analyzed NADPH oxidase activity and gene expression of its NOX2 and NOX4 subunits. As mitogen-activated protein kinases (MAPK) and the nuclear factor-kappa B (NF-*κ*B) pathway may be involved in skeletal muscle response to oxidative stress [21], we also evaluated protein expression of these pathways.

## 2. Materials and Methods

### 2.1. Experimental Animals and Study Protocol

Male Wistar rats weighing 90–100 g were purchased from the Central Animal House, Botucatu Medical School, UNESP. All experiments and procedures were approved by the Animal Experimentation Ethics Committee of Botucatu Medical School, UNESP, SP, Brazil, which follows the guidelines established by the Brazilian College for Animal Experimentation (protocol number 999/2013).

Rats were anaesthetized with a mixture of ketamine hydrochloride (50 mg/kg, i.m.) and xylazine hydrochloride (10 mg/kg, i.m.) and aortic stenosis (AS) was induced by placing a 0.6 mm stainless-steel clip on the ascending aorta via a thoracic incision according to a previously described method [19]. Sham operated rats were used as controls. All animals were housed in a temperature controlled room at 23°C and kept on a 12-hour light/dark cycle. Food and water were supplied* ad libitum*. Eighteen weeks after surgery, rats were subjected to transthoracic echocardiogram to evaluate degree of cardiac injury and assigned to four groups: sedentary control (C-Sed, *n* = 22), exercised control (C-Ex, *n* = 23), sedentary aortic stenosis (AS-Sed, *n* = 25), and exercised aortic stenosis (AS-Ex, *n* = 27) for eight weeks. At the end of the experimental period, rats were subjected to transthoracic echocardiogram and euthanized the next day. During euthanasia, we determined the presence or absence of clinical and pathologic heart failure features. The clinical finding suggestive of heart failure was tachypnea/labored respiration. Pathologic assessment of heart failure included pleuropericardial effusion, left atrial thrombi, ascites, hepatic congestion, pulmonary congestion (lung weight/body weight ratio higher than 2 standard deviations above the C-Sed group mean), and right ventricular hypertrophy (right ventricle weight/body weight ratio higher than 0.8 mg/g) [22, 23].

### 2.2. Exercise Testing

Functional capacity was assessed before and after the exercise period. Rats underwent 10 min/day testing environment adaption for one week before evaluations. Each animal was tested individually. The test consisted of an initial 5 min warm-up at 5 m/min on treadmill. The rats were then subjected to interval exercise at a speed of 8 m/min followed by 3 m/min increases in speed every 3 min until exhaustion. Exhaustion was determined when the animal refused to run even after electric stimulation or was unable to coordinate steps [24]. Maximum running speed was recorded and total distance calculated.

### 2.3. Exercise Training Protocol

Exercise was performed on a treadmill five times a week for eight weeks [14, 25]. There was an initial adaptation period, with a gradual increase in speed and exercise time. Speed from the 1st to the 3rd week was 5, 7.5, and 10 m/min and then remained constant until the end of the protocol. Exercise duration from the 1st to the 6th week was 10, 14, 18, 22, 26, and 30 min and then remained constant until the end of the experiment. In the first two weeks of training, animals were subjected to low-voltage electrical stimulation to start exercise. No animals were lost during exercise training.

### 2.4. Echocardiography

Cardiac structures and left ventricular function were evaluated by transthoracic echocardiogram and tissue Doppler imaging using a commercially available echocardiograph (General Electric Medical Systems, Vivid S6 model, Tirat Carmel, Israel) equipped with a 5–11.5 MHz multifrequency transducer as previously described [26–29]. After anesthesia with ketamine hydrochloride (50 mg/kg) and xylazine hydrochloride (1 mg/kg) intramuscularly, the rats were placed in the left lateral decubitus. A two-dimensional parasternal short-axis view of the left ventricle (LV) was obtained at the level of the papillary muscles. M-mode tracings were obtained from short-axis views of the LV at or just below the tip of the mitral-valve leaflets and at the level of the aortic valve and left atrium. M-mode images of the LV were printed on a black-and-white thermal printer (Sony UP-890MD) at a sweep speed of 100 mm/s. All LV structures were manually measured by the same observer (KO). Values obtained were the mean of at least five cardiac cycles on M-mode tracings. The following structural variables were measured: left atrium diameter (LA), LV diastolic and systolic diameters (LVDD and LVSD, resp.), LV diastolic (D) and systolic (S) posterior wall thickness (PWT) and septal wall thickness (SWT), and aortic diameter. Left ventricular mass (LVM) was calculated using the formula [(LVDD + DPWT + DSWT)^3^  − LVDD^3^] × 1.04. LV relative wall thickness (RWT) was calculated by the formula 2 × DPWT/LVDD. Left ventricular function was assessed by the following parameters: endocardial fractional shortening (EFS), midwall fractional shortening (MFS), ejection fraction (EF), posterior wall shortening velocity (PWSV), early and late diastolic mitral inflow velocities (E and A waves), E/A ratio, E-wave deceleration time (EDT), and isovolumetric relaxation time (IVRT). A joint assessment of diastolic and systolic LV function was performed using the myocardial performance index (Tei index). The study was complemented with evaluation by tissue Doppler imaging (TDI) of systolic (S′), early diastolic (E′), and late diastolic (A′) velocity of the mitral annulus (arithmetic average travel speeds of the lateral and septal walls) and E/E′ ratio.

### 2.5. Collection of Skeletal Muscle and Other Tissues for Analysis

Biological tissue collection was performed in the Experimental Research Laboratory (UNIPEX), Botucatu Medical School, UNESP. One day after final echocardiogram, the rats were weighed and anesthetized with intraperitoneal sodium pentobarbital (50 mg/kg) and euthanized. After blood collecting, hearts were removed by thoracotomy. Atria and ventricles were dissected and weighed separately. Soleus muscles of the right and left hind limbs were dissected, immediately weighed and frozen in liquid nitrogen, and stored at −80°C. Lung weight was used to assess the degree of pulmonary congestion. Fragments of lung and liver were weighed before and after drying sessions (65°C for 72 h) to evaluate wet/dry weight ratio.

### 2.6. Morphologic Study

Serial transverse 10 *μ*m thick sections of soleus muscles were cut in a cryostat cooled to −20°C and stained with hematoxylin and eosin. Measurements were performed using a microscope (Leica DM LS; Nussloch, Germany) attached to a computerized imaging analysis system (Media Cybernetics, Silver Spring, MD, USA). At least 150 cross-sectional fiber areas were measured from each soleus muscle.

### 2.7. Oxidative Stress Evaluation

#### 2.7.1. Antioxidant Enzymes Activity and Lipid Hydroperoxide Concentration

Soleus muscle samples (~200 mg) were homogenized in 5 mL of cold 0.1 M phosphate buffer, pH 7.0. Tissue homogenates were prepared in a motor-driven Teflon-glass Potter-Elvehjem, tissue homogenizer. The homogenate was centrifuged at 10,000 g, for 15 min at 4°C, and the supernatant was assayed for total protein, lipid hydroperoxide (LOOH), and glutathione peroxidase (GSH-Px, E.C.1.11.1.9), catalase (E.C.1.11.1.6.), and superoxide dismutase (SOD, E.C.1.15.1.1.) activities by spectrophotometry [7]. Enzyme activities were analyzed at 25°C using a microplate reader (*μ*Quant-MQX 200) with Kcjunior software for computer system control (Bio-Tec Instruments, Winooski, Vermont, USA). Spectrophotometric determinations were performed in a Pharmacia Biotech spectrophotometer with temperature-controlled cuvette chamber (UV/visible Ultrospec 5000 with Swift II applications software for computer system control, Cambridge, UK). All reagents were purchased from Sigma-Aldrich (St. Louis, MO, USA).

#### 2.7.2. Real-Time Quantitative Reverse Transcription-Polymerase Chain Reaction (RT-PCR)

Gene expression of NADPH oxidase subunits (NOX2, NOX4, p22^phox^, and p47^phox^) and reference genes cyclophilin and glyceraldehyde-3-phosphate dehydrogenase (GAPDH) was analyzed by RT-PCR according to a previously described method [30].

Total RNA was extracted from soleus muscles with TRIzol Reagent (Invitrogen Life Technologies, Carlsbad, CA, USA) and treated with DNase I (Invitrogen Life Technologies). One microgram of RNA was reverse transcribed using High Capacity cDNA Reverse Transcription Kit, according to standard methods (Applied Biosystems, Foster City, CA, USA). Aliquots of cDNA were then submitted to real-time PCR reaction using customized assay containing sense and antisense primers and Taqman (Applied Biosystems, Foster City, CA, USA) probes specific to each gene: NOX2 (Rn00576710_m1), NOX4 (Rn00585380_m1), p22^phox^ (Rn00577357_m1), and p47^phox^ (Rn00586945_m1). The amplification and analysis were performed using Step One Plus™ Real-Time PCR System (Applied Biosystems, Foster City, CA, USA). Data expression was normalized to reference gene expressions: cyclophilin (Rn00690933_m1) and GAPDH (Rn01775763_g1). Reactions were performed in triplicate and expression levels calculated using the CT comparative method (2^−ΔΔCT^).

#### 2.7.3. NADPH Oxidase Activity

NADPH oxidase activity was evaluated in membrane-enriched cellular fraction by reduction of lucigenin detected by luminometer. Soleus muscle was carefully washed in PBS to remove blood. Muscle fragments (~200 mg) were homogenized in 1 mL of ice-cold lysis buffer containing 50 mM Tris (pH 7.4), 100 mM DTPA, 0.1%  *β*-mercaptoethanol, and protease inhibitors. Samples were then sonicated (3 cycles of 10 s at 8 W) and centrifuged at 1,000 g for 3 min, at 4°C. Supernatant was transferred to another microtube and centrifuged at 18,000 g for 10 min, at 4°C, and transferred again to ultracentrifuge tubes and centrifuged at 100,000 g for 45 min, at 4°C. The supernatant was then discarded and the precipitate resuspended in 100 *μ*L of lysis buffer [31]. Total protein content was quantified by the Bradford method. Subsequently, 30 *μ*g of membrane-enriched cellular fraction was incubated in PBS (pH 7.4, containing EDTA 10 *μ*M) and 150 *μ*L of NADPH 2 mM in plastic tubes in a Berthold Sirius Luminometer, which performs automatic 20 *μ*L injections of 0.25 mM lucigenin into each tube. Luminescence data were collected every 2 s for 5 min using the FB12/Sirius program. NADPH oxidase activity was shown as the area under the curve of luminescence data obtained during the reaction.

#### 2.7.4. ROS Generation

Muscle fragment (~100 mg) was washed in PBS and incubated in a solution containing PBS/DTPA and 150 *μ*M dihydroethidium (DHE) for 25 min at 37°C in a dark room. The muscle fragment was then washed in PBS, transferred to liquid nitrogen, and homogenized with mortar and pestle. The homogenate was resuspended in acetonitrile (0.5 mL), sonicated (3 cycles at 8 W for 10 s), and centrifuged (12,000 g for 10 min at 4°C). Supernatant was dried under vacuum (Speed Vac Plus model SC-110A, Thermo Savant) and pellets were maintained at 20°C in the dark until analysis. Samples were resuspended in 80 *μ*L deionized water and injected into an HPLC system. Total ROS generation was evaluated by quantification of two DHE oxidation-derived fluorescent compounds, 2-hydroxyethidium (EOH) and ethidium, using the HPLC according to a previously described method [31, 32]. EOH is generated when DHE is oxidized by anion superoxide, while ethidium production is associated to heme proteins levels and peroxidase activity. DHE-derived products were expressed as ratios of generated EOH and ethidium over consumed DHE (initial DHE concentration minus remaining DHE).

### 2.8. Western Blotting

Soleus muscle protein levels were analyzed by Western blotting according to a previously described method [33] using specific anti-JNK (JNK 1/2 D-9 sc-137019), p-JNK (p-JNK G-7 sc-6254), p38 (p38 *α*/*β* A-12 sc-7972), p-p38 (p-p38 Thr 180/Tyr 182-R sc-17852-R), ERK (ERK 1 C-16 sc-93), p-ERK (p-ERK 1/2 Thr 202/Tyr 204 sc-16982), NF-*κ*B (p65 NF-*κ*B sc-7151), p-NF-*κ*B (Ser 536 p-p65 NF-*κ*B sc-33020), I*κ*B (I*κ*B-*α* sc-1643), and p-I*κ*B (p-I*κ*B-*α* sc-101713) antibodies (Santa Cruz Biotechnology, Santa Cruz, CA, USA). Protein levels were normalized to GAPDH (6C5 sc-32233, Santa Cruz Biotechnology).

Muscle protein was extracted using RIPA buffer and supernatant protein content was quantified by Bradford assay. Samples were separated on a polyacrylamide gel and then transferred to a nitrocellulose membrane. After blockade, membrane was incubated with the primary antibodies. Membrane was then washed with TBS and Tween 20 and incubated with secondary peroxidase-conjugated antibodies. Super Signal^*®*^ West Pico Chemiluminescent Substrate (Pierce Protein Research Products, Rockford, USA) was used to detect bound antibodies.

### 2.9. Statistical Analysis

Data are expressed as the mean ± standard deviation or median and percentiles. Comparisons between groups were performed by analysis of variance (ANOVA) for a 2 × 2 factorial design followed by the Tukey test or Mann-Whitney test (*p*  × 2 value). Comparisons of interest are as follows: C-Ex versus C-Sed, AS-Sed versus C-Sed, AS-Ex versus AS-Sed, and AS-Ex versus C-Ex. Mortality and frequency of heart failure features were compared between AS-Ex and AS-Sed groups using the Goodman test. The level of significance was set at 5%.

## 3. Results

### 3.1. Experimental Groups and Anatomic Parameters

At the end of the experimental period, C-Sed group contained 22 and C-Ex 19 rats. One rat from C-Sed had ascites and one from C-Ex presented pleural effusion. AS-Sed and AS-Ex groups had 18 rats each at the end of the experiment. Mortality did not statistically differ between groups. The frequency of heart failure features did not differ between AS-Ex and AS-Sed groups (Table 1).

Anatomical variables are presented in Table 2. Final body weight did not differ between groups. Left ventricle, right ventricle, atria, and lungs weights, absolute or normalized to body weight values, were higher in AS-Sed and AS-Ex than their respective controls. Soleus muscle weight was lower in the AS-Sed than C-Sed and gastrocnemius weight was lower in AS-Sed and AS-Ex than controls.

### 3.2. Exercise Testing

At initial exercise test, AS rats presented worse functional capacity than control groups, characterized by less run distance and time spent on the treadmill. At the end of the experiment, functional capacity was better in exercised than sedentary rats and worse in AS than control rats (Table 3).

### 3.3. Echocardiographic Evaluation

Before the exercise protocol, groups with aortic stenosis presented concentric left ventricular hypertrophy with mild systolic dysfunction and diastolic dysfunction. All parameters were similar in AS-Ex and AS-Sed groups (data not shown).

At the end of the experiment, structural variables did not differ between C-Ex and C-Sed group, except for reduced LV systolic posterior wall thickness in C-Ex. Both AS-Ex and AS-Sed presented higher LV systolic and diastolic diameters, LV wall thickness, relative wall thickness, aorta and left atrium diameters, and LV mass than their respective controls (Table 4). Functionally, there were no differences between C-Ex and C-Sed groups. AS-Ex and AS-C groups had decreased posterior wall shortening velocity, TDI systolic velocity of the mitral annulus, mitral A wave, isovolumetric relaxation time, and E wave deceleration time and increased mitral E wave, E/A ratio, and E wave/TDI early average mitral annulus diastolic velocity compared to respective controls. AS-Ex presented reduced endocardial and mesocardial fractional shortening and ejection fraction compared to C-Ex. We observed no differences between AS-Ex and AS-C groups (Tables 5 and 6).

### 3.4. Morphometric Analysis

Soleus muscle trophicity was evaluated on hematoxylin and eosin stained sections. Fiber cross-sectional area did not statistically differ between groups (C-Sed 3980 ± 231; C-Ex 4113 ± 253; AS-Sed 3541 ± 200, AS-Ex 3941 ± 253 *μ*m^2^; *p* > 0.05).

### 3.5. Oxidative Stress Evaluation

**Figure 1:**
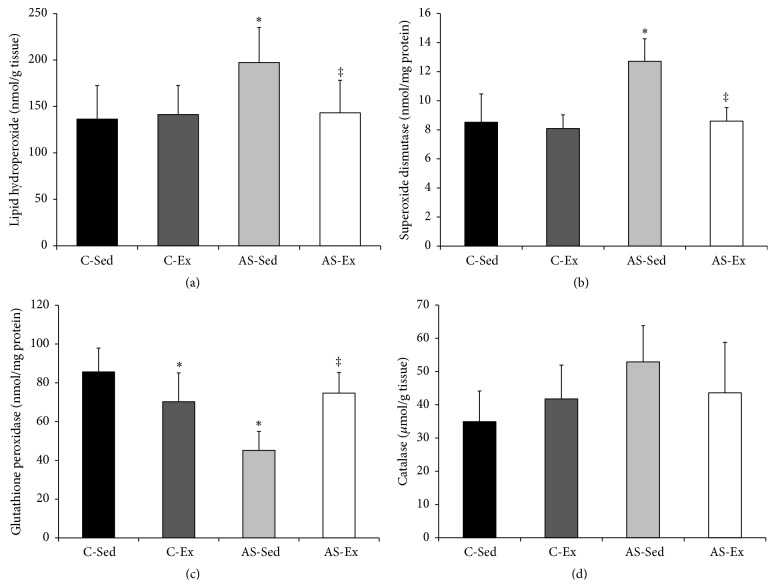
Lipid hydroperoxide concentration (a) and antioxidant enzymes activity in soleus muscle ((b) to (d)). C-Sed: sedentary control group; C-Ex: exercised control group; AS-Sed: sedentary aortic stenosis group; AS-Ex: exercised aortic stenosis group; *n*: number of animals. Data are mean ± SD (*n* = 8); ANOVA and Tukey; ^*∗*^ *p* < 0.05 versus C-Sed; ^‡^ *p* < 0.05 versus AS-Sed.

We used lipid hydroperoxide concentration as an oxidative stress biomarker in soleus muscle. The concentration was higher in AS-Sed than C-Sed and AS-Ex (C-Sed 136 ± 36.0; C-Ex 141 ± 31.2; AS-Sed 197 ± 37.8; AS-Ex 143 ± 35.0 nmol/g tissue; Figure 1(a)).

Glutathione peroxidase activity was lower in C-Ex and AS-Sed than C-Sed and higher in AS-Ex than AS-Sed (C-Sed 85.6 ± 12.3; C-Ex 70.2 ± 14.8; AS-Sed 45.2 ± 9.73; AS-Ex 74.7 ± 10.6 nmol/mg protein, *p* < 0.001; Figure 1(b)). Superoxide dismutase activity was higher in AS-Sed than C-Sed and AS-Ex (C-Sed 8.52 ± 1.95; C-Ex 8.09 ± 0.94; AS-Sed 12.7 ± 1.55, AS-Ex 8.60 ± 0.93 nmol/mg protein, *p* < 0.001; Figure 1(c)). Catalase activity did not differ between groups (C-Sed 34.9 ± 9.20; C-Ex 41.8 ± 10.2; AS-Sed 52.9 ± 10.9; AS-Ex 43.6 ± 15.2 *μ*mol/g tissue; Figure 1(d)).

**Figure 2:**
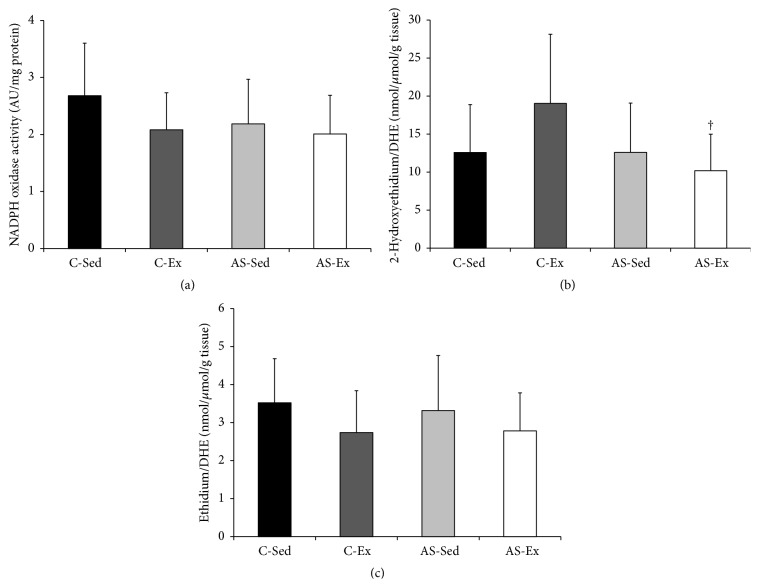
Soleus muscle NADPH oxidase activity analyzed by reduction of lucigenin (a), total reactive species generation by quantifying two dihydroethidium (DHE) oxidation-derived fluorescent compounds, 2-hydroxyethidium and ethidium, using HPLC ((b) and (c)). C-Sed: sedentary control group; C-Ex: exercised control group; AS-Sed: sedentary aortic stenosis group; AS-Ex: exercised aortic stenosis group; *n*: number of animals. Data are mean ± SD (*n* = 8); ANOVA and Tukey; ^†^ *p* < 0.05 versus C-Ex.

NADPH oxidase complex subunit p22^phox^ gene expression was higher in AS-Ex than C-Ex. Subunits p47^phox^, NOX2, and NOX4 gene expression did not differ between groups (Table 7). NADPH oxidase activity did not differ between groups (Figure 2(a)).

Total reactive oxygen species generation was evaluated in soleus muscle by quantification EOH and ethidium, two fluorescent compounds derived from DHE oxidation. The EOH/DHE ratio was lower in AS-Ex than C-Ex and did not differ between AS-Ex and AS-Sed (Figure 2(b)). The ethidium/DHE ratio did not differ between groups (Figure 2(c)).

### 3.6. Western Blotting

**Figure 3:**
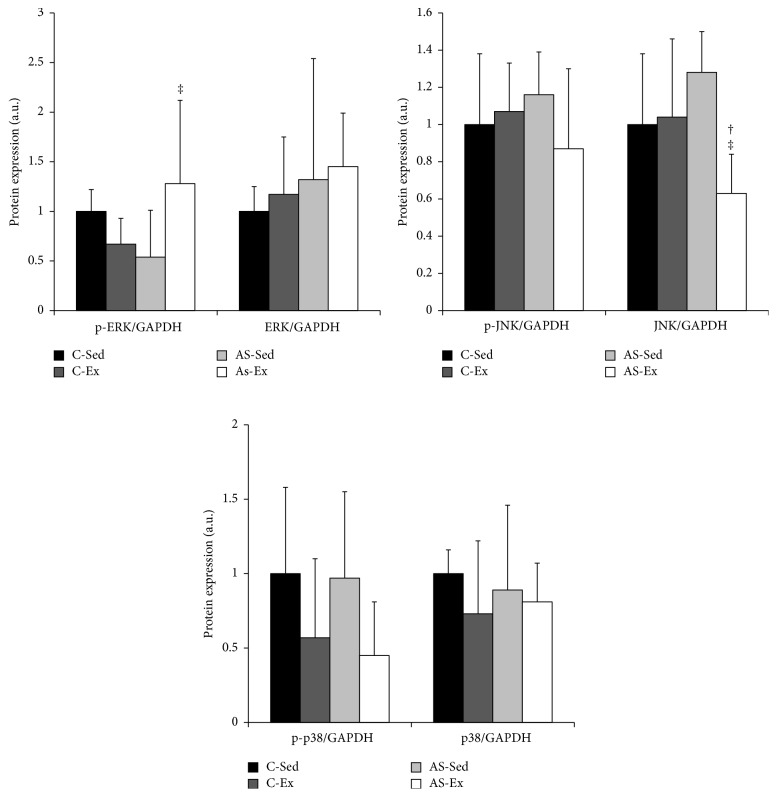
Protein levels of mitogen-activated protein kinases analyzed by Western blotting. Protein levels were normalized to GAPDH levels. C-Sed: sedentary control group; C-Ex: exercised control group; AS-Sed: sedentary aortic stenosis group; AS-Ex: exercised aortic stenosis group; *n*: number of animals. Data are mean ± SD (*n* = 7); ANOVA and Tukey; ^†^ *p* < 0.05 versus C-Ex; ^‡^ *p* < 0.05 versus AS-Sed.

MAPK protein expression is shown in Figure 3. Phosphorylated ERK was higher in AS-Ex than AS-Sed. Total JNK was lower in AS-Ex than C-Ex and AS-Sed. Protein expression of p38 did not differ between groups. Expression of total (p65 NF-*κ*B: C-Sed 1.00 ± 0.42, C-Ex 1.05 ± 0.34, AS-Sed 1.00 ± 0.65, and AS-Ex 0.83 ± 0.40) and phosphorylated [p-p65 NF-*κ*B: C-Sed 0.96 (0.87–1.06), C-Ex 0.99 (0.67–1.16), AS-Sed 0.71 (0.49–1.44), and AS-Ex 1.08 (0.76–1.17)] NF-*κ*B subunit, and total I*κ*B [C-Sed 0.97 (0.83–1.14), C-Ex 0.79 (0.68–0.87), AS-Sed 1.32 (1.13–1.84), and AS-Ex 1.53 (0.90–2.08)] did not differ between groups. Phosphorylated I*κ*B was lower in C-Ex and AS-Sed than in C-Sed (C-Sed 1.00 ± 0.21, C-Ex 0.71 ± 0.17, AS-Sed 0.54 ± 0.24, and AS-Ex 0.67 ± 0.39).

## 4. Discussion

In this study, we showed that physical exercise improves functional capacity in rats with aortic stenosis-induced heart failure regardless of changes in cardiac structures or left ventricular function. Additionally, we performed the first assessment of the influence of physical exercise on oxidative stress and MAPK and NF-*κ*B pathways in soleus muscle from aortic stenosis rats.

Aortic stenosis in young rats leads to early left ventricular hypertrophy, gradual ventricular dysfunction, and heart failure, similarly to human chronic pressure overload. After developing hypertrophy, rats remain compensated for approximately 20 to 28 weeks [19]. They then begin to present clinical and pathological heart failure features such as tachypnea, ascites, pleural effusion, atrial thrombus, lung congestion, and right ventricular hypertrophy. Left untreated, rats evolve to death within two to four weeks [34]. We therefore initiated the exercise protocol 18 weeks after aortic stenosis induction when no rat had tachypnea.

We used an exercise protocol adapted from studies with rats subjected to different models of cardiac injury such as myocardial infarction [35], arterial hypertension [14, 36], diabetes mellitus [25], and aortic stenosis [19]. All these conditions are characterized by a moderate-to-severe reduction in physical exercise tolerance. In spontaneously hypertensive rats [37, 38], voluntary wheel running, which is characterized by short periods of high intensity activity, has been associated with impaired cardiac remodeling [38]. Furthermore, untreated spontaneously hypertensive rats experienced sudden death at a running speed of 17.5 m/min [39]. We therefore used a low intensity aerobic exercise protocol, subjecting our rats to physical exercise at a maximum speed 10 m/min, a tolerable intensity for all rats.

Despite the low intensity of the aerobic exercise, it was effective in improving functional capacity of both control and aortic stenosis groups. At the end of the experiment, time on the treadmill and distance run were significantly higher in exercised than sedentary rats.

Body weight did not differ between groups. Decrease in body weight, known as cardiac cachexia, is often observed in advanced stages of heart failure in humans and is associated with poor prognosis, independently of important variables such as age, ejection fraction, exercise capacity, or functional class [40]. In this study, body weight preservation suggests that rats surviving to the end of the study were not in severe heart failure.

We performed transthoracic echocardiogram and tissue Doppler imaging before the exercise protocol to evaluate the degree of cardiac injury induced by aortic stenosis and assure homogeneity between groups. Both aortic stenosis groups presented concentric ventricular hypertrophy with mild systolic dysfunction and diastolic dysfunction. Cardiac remodeling with concentric hypertrophy and mild left ventricular dysfunction is usually observed in compensated chronic pressure overload [41].

After exercise, the aortic stenosis groups maintained the pattern of concentric left ventricular hypertrophy with slight ventricular dilatation and mild systolic dysfunction. Diastolic dysfunction was characterized by increased E/A ratio, which establishes the restrictive advanced pattern of diastolic dysfunction. We did not detect any significant differences between EA-Ex and AS-Sed groups. We therefore conclude that a light exercise protocol improves functional capacity without changing structural or functional heart parameters in aortic stenosis rats. The effects of exercise on the heart with aortic stenosis are not completely understood. Mice with mild or severe aortic stenosis subjected to voluntary rotating wheel exercise for eight weeks presented unchanged ventricular function with a trend towards aggravated LV dysfunction in severe aortic stenosis [18]. On the other hand, continuous treadmill aerobic exercise attenuated systolic dysfunction in aortic stenosis rats [19].

As cardiac function did not differ between AS-Ex and AS-Sed, the better physical capacity of exercised rats was probably related to functional improvement in skeletal muscles properties.

Skeletal muscles are composed of tissue which can change their metabolic, morphological, and functional characteristics after training or injury [42]. We evaluated the soleus, a muscle with predominantly slow twitch fibers and oxidative metabolism. Previous studies have shown that this muscle is sensitive to alterations induced by acute or chronic heart failure [6–8]. Furthermore, skeletal muscles preferentially composed of oxidative fibers are more sensitive to the effects of aerobic exercise than muscles preferably composed of glycolytic fibers [43].

We first evaluated redox status. Although the mechanisms involved in muscle alterations are not completely understood, it is well established that increased oxidative stress plays a role in muscle abnormalities [7, 44].

Lipid hydroperoxide concentration was greater in AS-Sed than C-Sed and AS-Ex, showing that muscle oxidative stress is increased during heart failure and normalized by exercise. Cellular response to oxidative stress depends on the intensity of the reactive oxygen species generation. At low concentrations, reactive oxygen species stimulates antioxidant enzymes and receptors; at high concentrations, however, they inhibit enzymatic activity leading to cellular damage [45]. In the AS-Sed group, increased oxidative stress was combined with increased superoxide dismutase activity and reduced glutathione peroxidase activity. Exercise prevented changes in antioxidant enzymes in the AS-Ex group. Reduction of antioxidant enzyme activity has been described in skeletal muscle during heart failure [7, 35, 44]. In addition, clinical and experimental studies have shown that exercise induces antioxidant properties in skeletal muscle during heart failure [35, 46]. Our results therefore show that aerobic exercise prevented increases in oxidative stress and changes in antioxidant enzymes.

The NADPH oxidase complex is an important source of reactive oxygen species. To determine whether this complex is involved in increased oxidative stress and its modulation by exercise, we evaluated NADPH oxidase activity and gene subunit expression. We analyzed gene expression of transmembrane subunits NOX2, NOX4, and p22^phox^ and the cytosolic subunit p47^phox^. NADPH oxidase activity and its subunits expression did not differ between groups, except for higher p22^phox^ subunit expression in AS-Ex than C-Ex. Despite this increased p22^phox^ gene expression, AS-Ex group NADPH oxidase activity was unchanged. We evaluated NADPH oxidase activity by measuring the light emitted by a reaction between reduced lucigenin and superoxide anion, which is a product of NADPH oxidase [31, 32]. The role of NADPH oxidase in muscle oxidative stress during heart failure is still unclear. In infarcted rats [21] and mice [47], increased NADPH oxidase activity was observed in the plantaris and hindlimb muscles. On the other hand, by evaluating NADPH oxidase activity using dihydroethidium, a molecule oxidized by superoxide anion, we found unchanged activity in soleus from rats with myocardial infarction-induced heart failure [7]. Different animal species, experimental model, or muscle evaluated may be responsible for divergent results.

Total reactive oxygen species generation was assessed by quantifying two fluorescent compounds derived from dihydroethidium oxidation: 2-hydroxyethidium and ethidium. Hydroxyethidium detection by HPLC fluorescence was proposed to more precisely measure superoxide production in blood vessels and was later adapted for use in striated muscle [31, 32]. Total reactive oxygen species generation did not change between groups, except for a lower hydroxyethidium/dihydroethidium ratio in AS-Ex than C-Ex. This result is in accordance with the lower lipid hydroperoxide concentration in the AS-Ex group.

Intracellular reactive oxygen species signaling pathways are not completely understood. Studies suggest that MAPK and the NF-*κ*B pathway are involved in muscle response to oxidative stress [21, 48]. MAPKs consist of four members: extracellular signal-regulated kinase (ERK) 1/2, p38, c-Jun NH2-terminal kinase (JNK), and ERK 5 [49]. Expression of the MAPK proteins did not differ between AS-Sed and C-Sed. Despite ERK expression being approximately 46% lower in AS-Sed than C-Sed, this difference did not reach statistical significance. Exercise reduced total JNK expression in AS-Ex compared to C-Ex and AS-Sed and increased phosphorylated ERK in AS-Ex compared to AS-Sed. While activation of JNK and p38 is associated with cell response to stress and muscle loss, activation of ERK is related to anabolic processes such as cell division, growth, and differentiation [49]. Therefore, in this study exercise modulated total JNK and phosphorylated ERK towards preserving protein synthesis and muscle mass. We have previously observed MAPK changes in diaphragm and soleus muscle in infarcted rats with heart failure [7, 50]. Except for reduced I*κ*B expression in C-Ex and AS-Sed compared to C-Sed, NF-*κ*B pathway protein expression remained unchanged in this experimental model.

## 5. Conclusion

Exercise improves the functional capacity of rats with aortic stenosis-induced heart failure regardless of changes in cardiac structures or left ventricular function. In soleus muscle, exercise reduces oxidative stress, preserves antioxidant enzyme activity, and modulates JNK and p-ERK expression with no changes in NADPH oxidase activity, NADPH oxidase subunits gene expression, or NF-*κ*B pathway protein expression.

## Figures and Tables

**Table 1:** Frequency of heart failure features in the aortic stenosis rats.

	Frequency (%)	
	AS-Sed (*n* = 18)	AS-Ex (*n* = 18)
Tachypnea/labored respiration	22.2	16.7
Left atrial thrombi	33.3	38.9
Pleuropericardial effusion	66.7	55.6
Hepatic congestion	16.7	11.1
Pulmonary congestion	66.7	66.7
Right ventricular hypertrophy	72.2	61.1
Ascites	22.2	5.60

AS-Sed: sedentary aortic stenosis group; AS-Ex: exercised aortic stenosis group; *n*: number of animals. Goodman test; *p* > 0.05.

**Table 2:** Anatomical data.

	C-Sed (*n* = 22)	C-Ex (*n* = 19)	AS-Sed (*n* = 18)	AS-Ex (*n* = 18)
BW (g)	454 ± 57.4	466 ± 64.6	435 ± 35.8	439 ± 47.8
LVW (mg)	0.92 (0.86–0.99)	0.96 (0.84–1.04)	1.40 (1.28–1.73)^*∗*^	1.36 (1.23–1.50)^†^
LVW/BW (mg/g)	1.95 (1.78–1.99)	1.88 (1.67–2.11)	3.17 (2.67–3.86)^*∗*^	3.35 (2.74–3.84)^†^
RVW (mg)	0.24 (0.21–0.25)	0.23 (0.21–0.28)	0.45 (0.31–0.55)^*∗*^	0.43 (0.26–0.54)^†^
RVW/BW (mg/g)	0.48 (0.42–0.51)	0.50 (0.41–0.56)	0.99 (0.76–1.22)^*∗*^	0.98 (0.62–1.28)^†^
Atria (mg)	0.09 (0.07–0.10)	0.09 (0.08–0.11)	0.31 (0.27–0.38)^*∗*^	0.27 (0.15–0.35)^†^
Atria/BW (mg/g)	0.19 (0.14–0.21)	0.19 (0.16–0.23)	0.68 (0.59–0.87)^*∗*^	0.68 (0.59–0.87)^†^
Lung (mg)	1.84 (1.65–2.02)	1.78 (1.48–2.03)	3.35 (2.31–4.08)^*∗*^	3.15 (2.11–3.50)^†^
Lung/BW (mg/g)	3.84 (3.54–4.25)	3.60 (2.98–4.12)	6.58 (4.40–8.10)^*∗*^	7.36 (4.83–10.25)^†^
Soleus (g)	0.25 ± 0.05	0.22 ± 0.05	0.22 ± 0.04^*∗*^	0.21 ± 0.03
Soleus/BW (mg/g)	0.50 ± 0.10	0.45 ± 0.11	0.48 ± 0.07	0.49 ± 0.09
Gastro (g)	2.67 ± 0.33	2.70 ± 0.29	2.44 ± 0.33^*∗*^	2.42 ± 0.27^†^
Gastro/BW (mg/g)	5.39 ± 0.41	5.51 ± 0.44	5.33 ± 0.54	5.54 ± 0.41

C-Sed: sedentary control group; C-Ex: exercised control group; AS-Sed: sedentary aortic stenosis group; AS-Ex: exercised aortic stenosis group; BW: body weight; LVW: left ventricle weight; RVW: right ventricle weight; Gastro: gastrocnemius muscle weight. Data are mean ± SD or median and percentiles; ANOVA and Tukey or Mann-Whitney; ^*∗*^ *p* < 0.05 versus C-Sed; ^†^ *p* < 0.05 versus C-Ex.

**Table 3:** Maximal exercise test before and after physical training.

	C-Sed (*n* = 22)	C-Ex (*n* = 19)	AS-Sed (*n* = 18)	AS-Ex (*n* = 18)
Distance				
Before	257 ± 86.5	247 ± 55.8	171 ± 59.7^*∗*^	163 ± 35.5^†^
After	225 ± 64.8	304 ± 47.5^*∗*^	154 ± 48.9^*∗*^	254 ± 47.2^†‡^

Time				
Before	22.8 ± 4.10	22.7 ± 2.53	18.5 ± 3.16^*∗*^	18.1 ± 2.16^†^
After	21.7 ± 3.51	25.1 ± 1.94^*∗*^	18.0 ± 3.05^*∗*^	22.7 ± 2.61^†‡^

C-Sed: sedentary control group; C-Ex: exercised control group; AS-Sed: sedentary aortic stenosis group; AS-Ex: exercised aortic stenosis group. Data are mean ± SD; ANOVA and Tukey; ^*∗*^ *p* < 0.05 versus C-Sed; ^†^ *p* < 0.05 versus C-Ex; ^‡^ *p* < 0.05 versus AS-Sed.

**Table 4:** Echocardiographic structural data.

	C-Sed (*n* = 22)	C-Ex (*n* = 19)	AS-Sed (*n* = 18)	AS-Ex (*n* = 18)
LVDD (mm)	8.30 ± 0.55	8.50 ± 0.55	9.02 ± 0.85^*∗*^	9.17 ± 0.74^†^
LVDD/BW (mm/kg)	16.6 ± 2.05	16.8 ± 1.91	18.9 ± 2.69^*∗*^	20.4 ± 3.09^†^
LVSD (mm)	4.05 ± 0.55	4.30 ± 0.60	4.81 ± 1.17^*∗*^	5.27 ± 0.98^†^
DPWT (mm)	1.42 (1.38–1.46)	1.38 (1.31–1.44)	2.06 (1.84–2.12)^*∗*^	1.99 (1.84–2.17)^†^
SPWT (mm)	3.01 (2.76–3.14)	2.69 (2.65–3.00)^*∗*^	3.60 (2.86–3.79)^*∗*^	3.49 (2.91–3.72)^†^
DSWT (mm)	1.43 (1.40–1.46)	1.38 (1.34–1.46)	2.11 (1.84–2.16)^*∗*^	2.01 (1.84–2.17)^†^
SSWT (mm)	2.52 (2.42–2.65)	2.42 (2.33–2.59)	2.79 (2.59–3.16)^*∗*^	2.93 (2.68–3.25)^†^
RWT	0.34 (0.33–0.36)	0.33 (0.31–0.34)	0.44 (0.38–0.48)^*∗*^	0.42 (0.40–0.50)^†^
AO (mm)	4.02 (3.83–4.16)	4.02 (3.83–4.16)	3.94 (3.80–4.16)	3.94 (3.80–4.09)
LA (mm)	5.29 (4.93–5.69)	5.66 (5.47–5.99)	8.54 (7.88–8.69)^*∗*^	8.14 (6.79–8.54)^†^
LA/AO	1.32 (1.26–1.38)	1.43 (1.30–1.52)	2.17 (1.83–2.29)^*∗*^	2.09 (1.67–2.24)^†^
LA/BW (mm/kg)	10.3 (9.5–12.1)	10.9 (10.0–12.3)	17.3 (14.8–19.5)^*∗*^	17.3 (15.7–18.9)^†^
LVM (g)	0.82 (0.77–0.89)	0.83 (0.74–0.93)	1.62 (1.18–1.87)^*∗*^	1.38 (1.32–1.93)^†^
LVMI (g/kg)	1.69 (1.44–1.88)	1.64 (1.47–1.85)	2.81 (2.69–3.67)^*∗*^	3.40 (2.66–4.16)^†^

C-Sed: sedentary control group; C-Ex: exercised control group; AS-Sed: sedentary aortic stenosis group; AS-Ex: exercised aortic stenosis group; LVDD and LVSD: left ventricular (LV) diastolic and systolic diameter, respectively; BW: body weight; DPWT and SPWT: LV diastolic and systolic posterior wall thickness, respectively; DSWT and SSWT: LV diastolic and systolic septal wall thickness, respectively; RWT: relative wall thickness; AO: aorta diameter; LA: left atrial diameter; LVM: LV mass; LVMI: LVM index. Data are mean ± SD or median and percentiles; ANOVA and Tukey or Mann-Whitney; ^*∗*^ *p* < 0.05 versus C-Sed; ^†^ *p* < 0.05 versus C-Ex.

**Table 5:** Echocardiographic evaluation of left ventricle systolic function.

	C-Sed (*n* = 22)	C-Ex (*n* = 19)	AS-Sed (*n* = 18)	AS-Ex (*n* = 18)
HR (bpm)	282 (262–318)	312 (255–341)	294 (280–325)	312 (290–318)
EFS %	51.6 (46.6–55.8)	50.0 (46.2–51.5)	46.6 (38.9–54.8)	43.6 (38.3–47.4)^†^
MFS %	29.5 (27.6–32.4)	29.7 (28.4–31.7)	28.6 (21.1–32.7)	24.5 (21.6–26.6)^†^
EF	0.89 (0.85–0.91)	0.87 (0.84–0.89)	0.85 (0.77–0.91)	0.82 (0.76–0.85)^†^
PWSV (mm/s)	41.1 ± 5.27	41.4 ± 7.10	31.0 ± 7.14^*∗*^	29.1 ± 6.66^†^
Tei index	0.44 ± 0.08	0.43 ± 0.07	0.41 ± 0.08	0.42 ± 0.11
TDI S′ (average, mm/s)	3.76 ± 0.72	3.58 ± 0.35	2.96 ± 0.69^*∗*^	2.94 ± 0.49^†^

C-Sed: sedentary control group; C-Ex: exercised control group; AS-Sed: sedentary aortic stenosis group; AS-Ex: exercised aortic stenosis group; HR: heart rate; EFS: endocardial fractional shortening; MFS: midwall fractional shortening; EF: ejection fraction; PWSV: posterior wall shortening velocity; Tei index: myocardial performance index; TDI S′: tissue Doppler imaging of systolic velocity of the mitral annulus. ANOVA and Tukey or Mann-Whitney; ^*∗*^ *p* < 0.05 versus C-Sed; ^†^ *p* < 0.05 versus C-Ex.

**Table 6:** Echocardiographic evaluation of left ventricle diastolic function.

	C-Sed (*n* = 22)	C-Ex (*n* = 19)	AS-Sed (*n* = 18)	AS-Ex (*n* = 18)
Mitral E (cm/s)	79.5 (73.0–84.0)	81.5 (76.0–89.0)	142 (89.0–165)^*∗*^	145 (85.0–159)^†^
Mitral A (cm/s)	59.5 (51.0–67.0)	60.0 (51.0–76.0)	24.0 (22.0–47.5)^*∗*^	22.5 (16.0–59.0)^†^
E/A	1.40 (1.22–1.53)	1.34 (1.17–1.60)	5.85 (1.73–7.15)^*∗*^	7.50 (1.47–9.16)^†^
IVRT (ms)	24.0 (22.0–26.0)	22.0 (21.2–26.0)	18.0 (15.0–22.0)^*∗*^	16.0 (15.0–22.0)^†^
IVRTn	53.0 ± 7.25	51.2 ± 8.37	42.6 ± 11.4^*∗*^	39.0 ± 10.2^†^
EDT (ms)	45.0 (41.0–52.0)	47.0 (41.0–53.0)	28.0 (23.0–35.0)^*∗*^	29.0 (24.5–33.0)^†^
TDI E′ (average mm/s)	4.37 (3.95–4.95)	4.50 (4.35–4.92)	4.25 (3.04–4.90)	4.17 (3.50–5.25)
TDI A′ (average mm/s)	4.50 ± 1.37	4.37 ± 1.21	3.79 ± 1.47	4.12 ± 1.07
E/TDI E′ (average)	17.9 (15.8–20.0)	18.4 (15.7–20.2)	33.0 (25.7–41.3)^*∗*^	32.1 (24.8–40.6)^†^

C-Sed: sedentary control group; C-Ex: exercised control group; AS-Sed: sedentary aortic stenosis group; AS-Ex: exercised aortic stenosis group. E/A: ratio between early- (E-) to-late (A) diastolic mitral inflow; IVRT: isovolumetric relaxation time; IVRTn: IVRT normalized to heart rate; EDT: E wave deceleration time; TDI E′ and A′: tissue Doppler imaging (TDI) of early (E′) and late (A′) diastolic velocity of mitral annulus. ANOVA and Tukey or Mann-Whitney; ^*∗*^ *p* < 0.05 versus C-Sed; ^†^ *p* < 0.05 versus C-Ex.

**Table 7:** Gene expression of NADPH oxidase subunits.

	C-Sed (*n* = 8)	C-Ex (*n* = 8)	AS-Sed (*n* = 8)	AS-Ex (*n* = 8)
p22^phox^	1.00 ± 0.40	0.44 ± 0.15	1.10 ± 0.66	1.17 ± 0.70^†^
p47^phox^	1.00 ± 0.53	1.52 ± 0.49	0.84 ± 0.48	1.21 ± 0.65
NOX2	1.00 (0.37–1.39)	0.33 (0.26–0.75)	0.82 (0.53–2.16)	0.63 (0.42–1.07)
NOX4	1.00 ± 0.52	0.70 ± 0.46	0.79 ± 0.22	0.98 ± 0.32

C-Sed: sedentary control group; C-Ex: exercised control group; AS-Sed: sedentary aortic stenosis group; AS-Ex: exercised aortic stenosis group. ANOVA and Tukey or Mann-Whitney; ^†^ *p* < 0.05 versus C-Ex.
